# Relationship between prehypertension and chronic kidney disease in middle-aged people in Korea: the Korean genome and epidemiology study

**DOI:** 10.1186/1471-2458-12-960

**Published:** 2012-11-09

**Authors:** Min-Ju Kim, Nam-Kyoo Lim, Hyun-Young Park

**Affiliations:** 1 Division of Cardiovascular and Rare Diseases, Center for Biomedical Science, National Institute of Health, Chungbuk, South Korea

**Keywords:** Blood pressure, Chronic kidney disease, Prehypertension, Estimated glomerular filtration rate

## Abstract

**Background:**

Elevated blood pressure (BP) is a major risk factor for the progression of chronic kidney disease (CKD). However, little is known about the influence of prehypertension on CKD. In this study, we investigated the relationship between prehypertension and CKD in a middle-aged Korean population. Furthermore, we prospectively evaluated the effect of active BP control on deterioration of kidney function during the two-year follow-up.

**Methods:**

The Korean Genome and Epidemiology Study is a community-based prospective cohort study started in 2001, with a follow-up survey conducted every two years. A total of 9509 participants aged 40–69 years were included in a baseline study. BP was classified according to the Seventh Report of the Joint National Committee on High BP (JNC-7) categories and CKD was defined as the presence of proteinuria or eGFR< 60mL/min/1.73m^2^. A multivariable logistic regression model was used to identify associations between BP and CKD.

**Results:**

The overall prevalence of CKD was 13.2%, and significantly increased with BP level. The multivariable-adjusted odds ratio of CKD was 1.59 for prehypertension and 2.27 for hypertension, compared with a normal BP. At the two-year follow-up, among the participants with prehypertension, subjects whose BP was poorly controlled had a significantly higher risk of eGFR drop (OR, 1.37; 95% CI, 1.13-1.67), as compared to controls. The prevalence of eGFR drop was 57.8% in the controlled BP group and 66.0% in the poorly-controlled BP group.

**Conclusions:**

Prehypertension, as well as hypertension, is significantly associated with CKD among middle-aged Koreans. Our results indicate that active control of the blood-pressure of prehypertensive individuals is needed to prevent deterioration of kidney function.

## Background

Chronic kidney disease (CKD) is associated with all-cause mortality, progression to end-stage renal disease (ESRD), cardiovascular disease (CVD), and many chronic diseases, such as diabetes [1-5].

The prevalence of CKD in the United States has increased from 10.0% in 1988–1994 to 13.1% in 1999–2004 [6]. In a population-based cross-sectional epidemiologic study in Korea, the prevalences of CKD and decreased kidney function were 13.7% and 5.0%, respectively [7]. According to the Kidney Disease Outcomes Quality Initiative (KDOQI) guidelines, CKD is defined as a marker of kidney damage and/or glomerular filtration rate (GFR) < 60 mL/min/1.73 m^2^ for at least three months; in addition, GFR is usually estimated from serum creatinine (Scr) values alone or prediction equations that take into account the Scr, age, gender and race [8]. Therefore, use of estimated glomerular filtration rate (eGFR) is commonly recommended to screen patients with CKD [8,9]. Furthermore, management of high risk patients who have hypertension and diabetes is particularly important [8,9].

Elevated blood pressure (BP) is closely associated with CKD progression, and lowering of BP may slow down GFR decline [10,11]. A number of studies have reported that hypertension is involved in CKD, emphasizing the importance of BP control in improving kidney function [12-14]. Both Kidney Early Evaluation Program (KEEP) and National Health and Nutrition Examination Survey (NHANES) data showed a strong association between hypertension and kidney disease [14]. The Seventh Report of the Joint National Committee on High BP (JNC-7) suggests controlling BP at <130/80 mmHg in patients with CKD [15]. Recently, a population-based, cross-sectional study from Singapore demonstrated that BP is independently associated with CKD, and that poorly controlled hypertension has a significantly stronger relationship with kidney damage than treated, controlled hypertension [16]. In addition, Higashikuni *et al*. found that a BP above 110/75 mmHg was significantly associated with albuminuria in a Japanese population without hypertension, but not with eGFR [17].

Although several studies have suggested that hypertension is a significant risk factor for CKD, little is known of its association with prehypertension and CKD. In this study, we investigated the relationship between prehypertension and CKD in a middle-aged Korean population. To determine the effect of BP control on deterioration of kidney function, we prospectively evaluated eGFR drop according to the change of BP during the two-year follow-up.

## Methods

### Study participants

**Figure 1:**
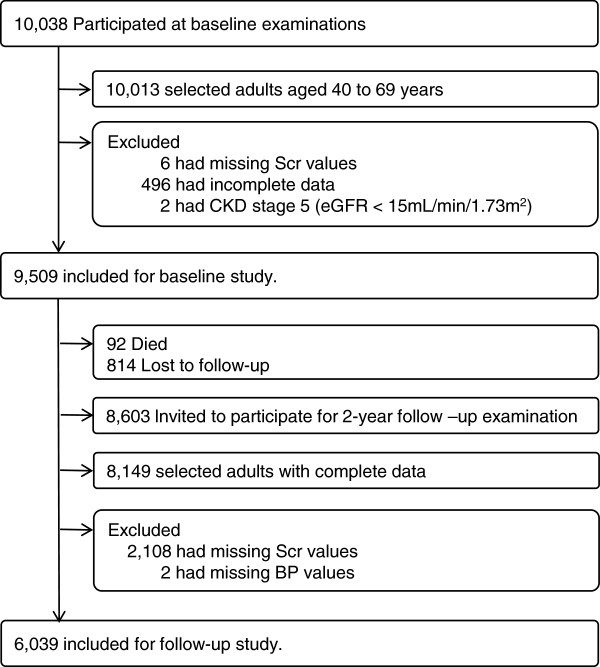
**Study participants at baseline and follow**-**up examinations.**

The Korean Genome and Epidemiology Study is a population-based prospective cohort study for the purpose of investigating the prevalence and risk factors of chronic disease in Korea, with the support of the Korean National Institute of Health. The baseline survey was performed in 2001–2002 on 10038 participants aged over 40 years and follow-up examinations were conducted every two year. Specimens were collected from the groups of residents representing each local population in a rural and an urban area (Anseong and Ansan, respectively). We obtained baseline and 2-year follow-up data from the Center for Genome Science in the National Institute of Health, Korea. The details of the present cohort have been described elsewhere [18,19]. Of 10038 participants enrolled in a rural and an urban area (Anseong and Ansan, respectively), 10013 subjects between the ages of 40 and 69 were selected to participate in a baseline survey. After exclusion of six subjects with missing Scr values, 496 who had incomplete data and two with CKD stage 5 (eGFR < 15 mL/min/1.73 m^2^), 9509 participants were included for baseline study. At the 2-year follow-up examination, 8603 participants were enrolled in this study after exclusion of 92 death and 814 lost to follow-up. Of those, 8149 people with complete data were selected to participate in a follow-up survey. We excluded 2108 and two participants with missing Scr and BP values, respectively. Thus, 6039 subjects were included in the present study (Figure 1). Of these, 2367 with prehypertension at baseline were selected and their BP change during two-year follow-up determined. Subjects were divided into controlled and poorly-controlled groups. The study protocol was approved by the Institutional Review Board of the Korea Centers for Disease Control and Prevention. Written informed consent was obtained from all study participants.

### Measurement of BP

Measurement of BP was performed after a 5 min period of rest using a mercury sphygmomanometer, and two BP readings were taken from both arms at 30 s intervals. The average systolic and diastolic BP values of two measurements taken in a sitting position were recorded.

BP was classified according to the Seventh Report of the Joint National Committee on High BP (JNC-7) categories [15]; normal BP (<120 mm Hg systolic and <80 mm Hg diastolic); prehypertension (120 to 139 mm Hg systolic or 80 to 89 mm Hg diastolic); hypertension (≥ 140 mm Hg systolic or ≥ 90 mm Hg diastolic systolic or use of antihypertensive medication).

Controlled BP was defined as participants who maintained a normal BP, and poorly-controlled BP as those with prehypertension or hypertension at the two-year follow-up.

### Other measurements

Demographic, medical and behavioral characteristics were obtained using a standardized questionnaire, and included age, sex, cigarette smoking and alcohol intake.

Body weight and height were measured to the nearest 0.1 kg or 0.1 cm and body mass index (BMI) was calculated as body weight (kg) divided by height (meters) squared. Waist circumference (WC) was measured at the midpoint between the ribs and the iliac crest in the standing position.

Blood samples were collected after an 8–14 h fast. Fasting plasma glucose (FPG), total cholesterol (TC), triglyceride (TG), high-density lipoprotein cholesterol (HDL-C) and Scr levels were measured enzymatically, and low-density lipoprotein cholesterol (LDL-C) levels were estimated by the Friedwald formula [20]. Proteinuria tested by the dipstick method was defined as 1 + or more.

### Definitions of CKD and diabetes mellitus (DM)

According to the Kidney Disease Outcomes Quality Initiative (KDOQI) guidelines, CKD is defined as a marker of kidney damage and/or GFR < 60 mL/min/1.73 m^2^ for at least three months [8]. We defined CKD as the presence of proteinuria or eGFR < 60 mL/min/1.73 m^2^. CKD stages were classified as follows: stage 1, proteinuria (≥ 1 +) with eGFR ≥ 90 mL/min/1.73 m^2^; stage 2, proteinuria (≥1 +) with eGFR 60–89 mL/min/1.73 m^2^; stage 3, eGFR 30–59 mL/min/1.73 m^2^, and stage 4, eGFR 15–29 mL/min/1.73m^2^. eGFR was calculated using the following equation [8]: eGFR = 186.3 × (Scr in mg/dL)^-1.154^ × Age^- 0.203^ × (0.742 if female). Changes in kidney function were evaluated using baseline and two-year follow-up eGFR values. eGFR drop denotes a decline in eGFR between baseline and the two-year follow-up.

DM was diagnosed as an FPG ≥126 mg/dl or 2-h post-challenge plasma glucose (2 h PG) ≥200 mg/dl or hemoglobin A_1C_ (HbA_1C_) ≥6.5%, or use of oral hypoglycemic agents using the 2010 criteria of the American Diabetes Association (ADA) [21].

### Statistical analysis

The baseline characteristics of subjects were expressed as means and standard deviations for continuous variables. Statistical differences between groups were performed by one-way analysis of variance (ANOVA) for continuous variables and the chi-square test for categorical variables. A multivariable logistic regression model was used to identify associations between BP and CKD. C-statistics were calculated to compare the ability of different logistic models to discriminate participants with and without CKD. After two-year follow-up, the odds ratio of eGFR drop according to BP change was calculated by multivariable logistic regression analysis. Values of *P* <0.05 were considered to be statistically significant. The data were analyzed using the SAS software (version 9.2; SAS Institute, Cary, North Carolina).

## Results

### Baseline characteristics

The baseline characteristics of the participants according to BP category are shown in Table 1. Of 9509 participants, there were 3792 normotensive (39.9%), 3873 prehypertensive (40.7%) and 1844 hypertensive (19.4%) subjects. The mean age of subjects was 52 years, 47.9% were male and their mean BMI was 24.6 kg/m^2^. The BMI, WC, FPG, TC, LDL-C and TG levels were increased when BP level was high. Hypertensive participants were more likely to have a history of DM (11.5% in the prehypertension group and 19.3% in the hypertension group). Of all subjects, 25.8% were current smokers and 47.7% were alcohol drinkers. The mean GFR estimated using the Modification of Diet in Renal Disease (MDRD) equation was 74.0 ± 14.0 mL/min/1.73 m^2^, and was lowest in the hypertension group. The proportion of individuals with eGFR <60 mL/min/1.73 m^2^ in the normal BP, prehypertension and hypertension groups were 5.6%, 11.4% and 23.3%, respectively. Two-hundred and seventeen subjects (2.3%) had proteinuria (≥1+), which was more prevalent in subjects with hypertension than in those with a normal BP.

**Table 1:** Mean values of baseline characteristics according to BP category

**Variables**	**Total (*n***=**9509)**	**BP category**			***P***-**value**
		**Normal BP (*n***=**3792)**	**Prehypertension (*n***=**3873)**	**Hypertension (*n***=**1844)**	
Age (years)	52.1 ± 8.9	49.0 ± 7.9	52.8 ± 8.8	56.9 ± 8.2	<0.001
40-49	4557 (47.9)	2393 (63.1)	1730 (44.7)	434 (23.5)	
50-59	2481 (26.1)	847 (22.3)	1042 (26.9)	592 (32.1)	
60-69	2471 (26.0)	552 (14.6)	1101 (28.4)	818 (44.4)	
Gender, male	4566 (48.0)	1594 (42.0)	2164 (55.9)	808 (43.8)	<0.001
BMI (kg/m^2^)	24.6 ± 3.1	23.8 ± 2.9	24.7 ± 3.1	25.8 ± 3.3	<0.001
WC (cm)	82.6 ± 8.8	79.5 ± 8.2	83.6 ± 8.4	86.7 ± 8.5	<0.001
Diabetes	1069 (11.2)	267 (7.0)	447 (11.5)	355 (19.3)	<0.001
Current smokers	2455 (25.8)	960 (25.3)	1125 (29.1)	370 (20.1)	<0.001
Alcohol drinkers	4535 (47.7)	1715 (45.2)	2016 (52.1)	804 (43.6)	<0.001
SBP (mmHg)	121.3 ± 18.4	105.5 ± 8.2	125.9 ± 9.9	144.4 ± 17.6	<0.001
DBP (mmHg)	80.3 ± 11.4	70.2 ± 6.0	84.2 ± 6.5	92.7 ± 10.7	<0.001
FPG (mg/dL)	87.3 ± 21.4	84.7 ± 19.1	88.1 ± 21.3	91.3 ± 25.3	<0.001
TC (mg/dL)	191.5 ± 35.7	186.6 ± 34.1	193.2 ± 35.7	198.3 ± 37.3	<0.001
HDL-C (mg/dL)	44.8 ± 10.1	45.3 ± 9.9	44.7 ± 10.1	43.9 ± 10.3	<0.001
LDL-C (mg/dL)	114.6 ± 33.6	113.2 ± 31.5	114.6 ± 34.2	117.4 ± 36.0	<0.001
TG (mg/dL)	161.0 ± 103.0	140.5 ± 89.9	169.9 ± 109.9	184.7 ± 105.3	<0.001
Scr (mg/dL)	1.00 ± 0.10	0.99 ± 0.10	1.00 ± 0.11	1.00 ± 0.20	<0.001
eGFR (mL/min/1.73 m ^2^)	74.0 ± 14.0	73.9 ± 14.0	75.2 ± 13.8	71.6 ± 13.9	<0.001
≥90	523 (5.5)	219 (5.8)	210 (5.4)	94 (5.1)	
60-89	7903 (83.1)	3361 (88.6)	3222 (83.2)	1320 (71.6)	
30-59	1073 (11.3)	212 (5.6)	439 (11.3)	422 (22.9)	
15-29	10 (0.1)	0 (0.0)	2 (0.1)	8 (0.4)	
Proteinuria(≥1+)	217 (2.3)	45 (1.2)	92 (2.4)	79 (4.3)	<0.001

Data are expressed as mean ± SD or percentage. One-way ANOVA was used for continuous variables.

BP categories were defined as follows: Normal BP, SBP <120 mmHg and DBP <80 mmHg; Prehypertension, SBP 120–139 mmHg or DBP 80–89 mmHg; Hypertension, SBP ≥140 mmHg or DBP ≥90 mmHg or use of antihypertensive medications.

### Prevalence of CKD

The overall prevalence of CKD stages 1 to 4 was 13.2%, and increased significantly with BP level (*P* trend <0.001). CKD prevalence according to stage was 13.8% in stages 1 and 2, 85.4% in stage 3, and 0.8% in stage 4. Participants with hypertension showed the highest CKD prevalence. There was a gender difference in CKD prevalence (3.5% in males and 22.2% in females). Females had a higher prevalence of CKD in all categories (Table 2).

**Table 2:** Prevalence of CKD according to BP category

**Variables**	**Total**	**BP category**			***P***-**value**
		**Normal BP (*n***=**3792)**	**Prehypertension (*n***=**3873)**	**Hypertension (*n***=**1844)**	
All (*n*=9509)					
No-CKD	8252 (86.8)	3541 (93.4)	3357 (86.7)	1354 (73.4)	<0.001
All CKD	1257 (13.2)	251 (6.6)	516 (13.3)	490 (26.6)	
Stages 1 and 2	174 (13.8)	39 (15.5)	75 (14.5)	60 (12.2)	
Stage 3	1073 (85.4)	212 (84.5)	439 (85.1)	422 (86.1)	
Stage 4	10 (0.8)	0 (0.0)	2 (0.4)	8 (1.6)	
Males (*n*=4566)					
No-CKD	4408 (96.5)	1571 (98.6)	2088 (96.5)	749 (92.7)	<0.001
All CKD	158 (3.5)	23 (1.4)	76 (3.5)	59 (7.3)	
Stages 1 and 2	111 (70.3)	20 (87.0)	53 (69.7)	38 (64.4)	
Stage 3	44 (27.8)	3 (13.0)	23 (30.3)	18 (30.5)	
Stage 4	3 (1.9)	0 (0.0)	0 (0.0)	3 (5.1)	
Females (*n*=4943)					
No-CKD	3844 (77.8)	1970 (89.6)	1269 (74.3)	605 (58.4)	<0.001
All CKD	1099 (22.2)	228 (10.4)	440 (25.8)	431 (41.6)	
Stages 1 and 2	63 (5.7)	19 (8.3)	22 (5.0)	22 (5.1)	
Stage 3	1029 (93.6)	209 (91.7)	416 (94.5)	404 (93.7)	
Stage 4	7 (0.6)	0 (0.0)	2 (0.5)	5 (1.2)	

Data are expressed as *n* (%) and tested by chi-square test at *P* < 0.05.

CKD was defined as estimated glomerular filtration rate (eGFR) <60 ml/min per 1.73 m^2^ and/or the presence of proteinuria (≥1+).

### Results of multivariable logistic regression analysis of CKD

The results of multivariable logistic regression analysis of CKD according to BP category are presented in Table 3. The multivariable-adjusted odds ratio (OR) of CKD was 1.59 (95% CI, 1.29-1.95) in subjects with prehypertension, and 2.27 (95% CI, 1.80-2.86) in subjects with hypertension, compared with those with a normal BP. Both prehypertension and hypertension showed significant positive associations with CKD after adjustment for age, sex, BMI, FPG, TC, TG, HDL-C, WC, and smoking and alcohol drinking habits. The c-statistic was 0.90 in the multivariable-adjusted Model 3, which had a better discrimination capability than the unadjusted model (c-statistic of 0.66).

**Table 3:** **Unadjusted and multivariable**-**adjusted odds ratio** (**OR**) **and c statistics of CKD according to BP category**

**Variables**	**CKD**			
	**Unadjusted OR (95% CI)**	**Model 1 OR (95% CI)**	**Model 2 OR (95% CI)**	**Model 3 OR(95% CI)**
Normal BP	1.00 (reference)	1.00 (reference)	1.00 (reference)	1.00 (reference)
Prehypertension	2.17 (1.85-2.54)^*^	1.69 (1.38-2.06)^*^	1.68 (1.37-2.06)^*^	1.59 (1.29-1.95)^*^
Hypertension	5.11 (4.33-6.02)^*^	2.52 (2.02-3.13)^*^	2.49 (1.99-3.12)^*^	2.27 (1.80-2.86)^*^
c-statistic	0.66	0.89	0.89	0.90

Model 1: adjusted for age and sex.

Model 2: adjusted for age, sex and BMI.

Model 3: adjusted for age, sex, BMI, FBG, TC, TG, HDL-C, WC, current smokers and alcohol drinkers.

^*^P <0.001.

### Odds ratio for eGFR drop during the two-year follow-up

Table 4 shows the odds ratio (OR) for eGFR drop according to the change of BP during the two-year follow-up. Among all subjects included in the follow-up study, 3133 had controlled BP (normal BP at two-year follow-up) and 2906 had poorly-controlled BP (prehypertension or hypertension at two-year follow-up). A total of 1644 (52.5%) subjects in the controlled and 1827 (62.9%) in the poorly-controlled BP groups experienced a drop in eGFR. According to the multivariable logistic regression analysis, subjects whose BP was not controlled had a significantly higher risk of eGFR drop (OR, 1.22; 95% CI, 1.08-1.38) when the controlled BP group was used as a reference. Of 2367 subjects with prehypertension, 857 had controlled BP (normal BP at two-year follow-up) and 1510 had poorly-controlled BP (prehypertension or hypertension at two-year follow-up). The prevalence of eGFR drop was 57.8% in the controlled BP group and 66.0% in the poorly-controlled BP group. Compared to subjects with controlled BP, the risk of eGFR drop increased by 1.37 (95% CI, 1.13-1.67) times for those with poorly-controlled BP after adjustment for age, sex, BMI, FPG, TC, TG, HDL-C, WC, and smoking and alcohol drinking habits. The c-statistics in multivariable-adjusted Model 3 showed the best discrimination capability.

**Table 4:** **Unadjusted and multivariable**-**adjusted odds ratio** (**OR**) **for eGFR drop according to change in BP**

**BP change after 2 years**	**No**. **at risk**	**No**. **with eGFR drop (%)**^**a**^	**eGFR drop**			
			**Unadjusted OR (95% CI)**	**Model 1 OR (95% CI)**	**Model 2 OR (95% CI)**	**Model 3 OR (95% CI)**
All						
**Controlled BP**^**b**^	3133	1644 (52.5)	1.00 (reference)	1.00 (reference)	1.00 (reference)	1.00 (reference)
**Poorly**-**controlled BP**^**c**^	2906	1827 (62.9)	1.53 (1.38-1.70)^**^	1.31 (1.16-1.47)^**^	1.23 (1.09-1.39)^**^	1.22 (1.08-1.38)^*^
c-statistic			0.55	0.74	0.75	0.76
Prehypertension						
**Controlled BP**^**b**^	857	495 (57.8)	1.00 (reference)	1.00 (reference)	1.00 (reference)	1.00 (reference)
**Poorly**-**controlled BP**^**c**^	1510	997 (66.0)	1.42 (1.20-1.69)^**^	1.39 (1.15-1.69)^**^	1.35 (1.11-1.63)^*^	1.37 (1.13-1.67)^*^
c-statistic			0.54	0.74	0.75	0.76

^a^eGFR drop denotes the decline in eGFR between baseline and the two-year follow-up.

(Changes in eGFR (Δ) = eGFR _at two-year follow-up_ – eGFR _at baseline_).

^b^Controlled BP was defined as participants who had a normal BP at the two-year follow-up.

^c^Poorly-controlled BP was defined as participants who were prehypertensive or hypertensive at the two-year follow-up.

Model 1: adjusted for age and sex.

Model 2: adjusted for age, sex and BMI.

Model 3: adjusted for age, sex, BMI, FBG, TC, TG, HDL-C, WC, current smokers and alcohol drinkers.

^*^P<0.01, ^**^P<0.001.

## Discussion

The purpose of this study was to examine the relationship between BP and CKD in a middle-aged Korean population. We found a strong and independent association between prehypertension, as well as hypertension, and CKD. Our data also indicated that the risk of eGFR drop at the two-year follow-up was significantly greater when BP was poorly-controlled. The risk increased beginning with prehypertensive participants.

In this study of 9509 Koreans aged 40–69 years, the prevalence of CKD was 13.2%, similar to the results of an epidemiologic study in urban Korea, which reported an overall CKD prevalence of 13.7% [7]. The prevalence of CKD was greater in females than in males (3.5% in males and 22.2% in females) as in other Korean population studies [22]. This discrepancy may be possibly explained by the increased filtration fraction in men, which may contribute to maintain the GFR [23,24].

In addition, results from NHANES 1999–2004 in the United States indicated that the prevalence of CKD stages 1 to 4 was 13.1% [6]. Among Australian adults aged 25 years or older, approximately 16% had at least one indicator of kidney damage [25]. The prevalence of CKD differs among ethnic groups, and the Asian population had a relatively high prevalence [26]. It is estimated that, in Japan, approximately 19% of the adult population has stages 3 to 5 CKD [27], and the prevalence of CKD in Beijing was 12.9% [28]. Furthermore, in the NHANES 1999–2006 in the US, 17.3% and 22.0% of those with prehypertension and undiagnosed hypertension, respectively, had CKD as determined by eGFR or albuminuria [29].

We used the MDRD equation to estimate GFR because it has been widely used in epidemiologic studies and clinical practice. When eGFR was calculated by the Chronic Kidney Disease Epidemiology Collaboration (CKD-EPI) equation [30], the mean eGFR and prevalence of CKD was similar to our results [see Additional file 1, Additional file 2. In addition, when we applied the equations for Korean population [31], the mean eGFR differed from those by the MDRD equation and the prevalence of CKD was lower than in our results, especially in females [see Additional file 1, Additional file 3, Additional file 4. However, the equations for Korean population may need to be evaluated its validity in a variety of population with larger sample size.

We found that CKD prevalence increased with BP level; normotensive subjects showed the lowest prevalence and hypertensive subjects the highest (6.6, 13.3 and 26.6% for normal BP, prehypertension and hypertension, respectively).

Hypertension is a well-known risk factor for CKD progression [12-14]. However, the influence of BP on CKD in the general population in Korea has not been thoroughly evaluated. In addition, whether prehypertension has an effect on CKD remains controversial. In this study, we focused particularly on the risk of CKD in prehypertensive subjects.

Several cohort studies provided strong epidemiologic evidence regarding the risk of ESRD across the BP categories. Our results were consistent with those of an earlier study, which reported that the risk of ESRD was increased even with normal but not optimal and high-normal levels, as well as hypertension [32,33]. In our data, a multiple logistic regression analysis indicated that prehypertensive and hypertensive participants had a ~1.6 and ~2.3 times increased risk of CKD. Moreover, studies from the Asia-Pacific region reported that high BP was the strongest risk factor (>80% greater) for renal death, indicating the importance of risk factors for CKD in Asia [34]. Recent studies of the combined effect of prehypertension and obesity on CKD risk showed that prehypertensive and obese subjects were at increased risk of CKD [35,36].

Our follow-up results showed that subjects with poorly-controlled BP were more likely to have a decreased eGFR compared with those with controlled BP. The risk of eGFR drop was significantly increased among the poorly-controlled BP group in all, and prehypertensive participants (Table 4). These findings support the JNC-7 recommendation: a reduction in BP to <130/80 mmHg for CKD patients to prevent cardiovascular disease outcomes [15]. The MDRD trial demonstrated a beneficial effect of BP control on progression of chronic kidney disease; this may support our findings. In a long-term follow-up of participants with a moderately- to severely-decreased GFR, as compared to baseline, those with a reduced target BP had a delayed onset of kidney failure and the composite outcome, as compared with the usual-target group [37]. Also, effective control of BP using antihypertensive agents such as angiotensin-converting enzyme inhibitors (ACEI), reduced proteinuria and ameliorated GFR [38].

Interestingly, the risk of CKD significantly increased in prehypertensive subjects, and those with poorly controlled BP had a higher risk of eGFR drop at the two-year follow-up. That is, the increased CKD risk begins at a near-normal BP; therefore, those individuals may be considered for active BP control to reduce the burden of kidney disease.

The strength of this study was that it was a prospective community-based cohort study which evaluated the association between BP and CKD across the range of BP.

However, there were also some limitations to this study. First, the study population consisted only of those aged 40–69 years. Nevertheless, our results may be representative of the general population in Korea because the data were from a community-based prospective cohort study, which may have minimized the sampling bias effect. Second, Scr and proteinuria were measured only once. According to the K/DOQI guidelines, CKD is defined as a GFR <60 mL/min/1.73 m^2^ or kidney damage for at least three months; therefore, Scr and proteinuria should be evaluated more than once. Thus, there might be an underestimation or overestimation of the prevalence of CKD in both males and females. Third, the MDRD equation has not been validated in the Korean population. Therefore, eGFR calculation using the MDRD equation might be inaccurate. Fourth, each participant’s BP measurement was based on only two readings of a single day. Also, we only evaluated in a state of BP control with BP values and self-reported medication at a baseline and 2-year follow-up. Therefore, the misclassification of BP may be possible and it might be underestimated or overestimated the prevalence of controlled BP and poorly-controlled BP.

## Conclusions

Prehypertension, as well as hypertension, is significantly associated with CKD among middle-aged Koreans. Our results indicate that active control of the blood-pressure of prehypertensive individuals is necessary to prevent deterioration in kidney function. Therefore, we suggest that active blood-pressure control may play an important role in prevention of CKD.

## Abbreviations

BP: Blood Pressure ; CKD: Chronic Kidney Disease ; ESRD: End-Stage Renal Disease ; CVD: CardioVascular Disease ; KDOQI: Kidney Disease Outcomes Quality Initiative ; eGFR: Estimated Glomerular Filtration Rate ; Scr: Serum creatinine ; KEEP: Kidney Early Evaluation Program ; NHANES: National Health and Nutrition Examination Survey ; BMI: Body Mass Index ; WC: Waist Circumference ; FPG: Fasting Plasma Glucose ; TC: Total Cholesterol ; TG: Triglyceride ; HDL-C: High-Density Lipoprotein Cholesterol ; LDL-C: Low-Density Lipoprotein Cholesterol ; DM: Diabetes Mellitus ; 2 h PG: 2-h Post-challenge plasma Glucose ; HbA_1C_: Hemoglobin A_1C_; ADA: American Diabetes Association ; ANOVA: One-way Analysis Of Variance ; MDRD: Modification of Diet in Renal Disease ; SBP: Systolic Blood Pressure ; DBP: Diastolic Blood Pressure .

## Competing interests

The authors declare that they have no competing interests.

## Authors’ contributions

MJK and HYP participated in the design of the study, performed the statistical analysis and participated in drafting the manuscript. NKL performed the statistical analysis. All authors read and approved the final manuscript.

## Pre-publication history

The pre-publication history for this paper can be accessed here:

http://www.biomedcentral.com/1471-2458/12/960/prepub

## Supplementary Material

Additional file 1 Mean GFR according to different estimating equations. Click here for file

Additional file 2 Prevalence of CKD according to the CKD-EPI equation. Click here for file

Additional file 3 Prevalence of CKD according to the MDRD equation with the Korean coefficient. Click here for file

Additional file 4 Prevalence of CKD according to the novel equation for Korean. Click here for file
