# Supplementary material for: Relationship between prehypertension and chronic kidney disease in middle-aged people in Korea: the Korean genome and epidemiology study
Source: BMC Public Health. 2012 Nov 9;12:960. doi: 10.1186/1471-2458-12-960 (PMC3549294; doi:10.1186/1471-2458-12-960)
Supplement: Additional file 1 — Mean GFR according to different estimating equations. [file 1471-2458-12-960-S1.doc]

**Additional file 1.** Mean GFR according to different estimating equations.

| **Variables** | **Total**  **(*n*=9509)** | | | **BP category** | | | | | | | | |
| --- | --- | --- | --- | --- | --- | --- | --- | --- | --- | --- | --- | --- |
|  |  |  |  | **Normal BP**  **(*n*=3792)** | | | **Prehypertension**  **(*n*=3873)** | | | **Hypertension**  **(*n*=1844)** | | |
| **eGFR _MDRD_ (mL/min/1.73m^2^)** |  |  |  |  |  |  |  |  |  |  |  |  |
| All | 74.0 | ± | 14.0 | 73.9 | ± | 14.0 | 75.2 | ± | 13.8 | 71.6 | ± | 13.9 |
| Males | 82.7 | ± | 9.4 | 83.5 | ± | 9.1 | 82.7 | ± | 9.2 | 81.1 | ± | 10.3 |
| Females | 65.9 | ± | 12.6 | 67.0 | ± | 12.6 | 65.7 | ± | 12.9 | 64.1 | ± | 11.6 |
| **eGFR _CKD-EPI_ (mL/min/1.73m^2^)** |  |  |  |  |  |  |  |  |  |  |  |  |
| All | 76.3 | ± | 13.0 | 77.0 | ± | 12.6 | 77.3 | ± | 12.9 | 72.8 | ± | 13.2 |
| Males | 84.8 | ± | 8.9 | 86.2 | ± | 8.4 | 84.7 | ± | 8.6 | 82.2 | ± | 10.1 |
| Females | 68.4 | ± | 11.0 | 70.3 | ± | 10.8 | 67.8 | ± | 11.0 | 65.4 | ± | 10.4 |
| **eGFR _MDRD with the Korean coefficient_ (mL/min/1.73m^2^)** |  |  |  |  |  |  |  |  |  |  |  |  |
| All | 81.2 | ± | 15.3 | 81.2 | ± | 15.3 | 82.6 | ± | 15.2 | 78.6 | ± | 15.3 |
| Males | 90.8 | ± | 10.3 | 91.7 | ± | 10.0 | 90.8 | ± | 10.1 | 89.1 | ± | 11.3 |
| Females | 72.4 | ± | 13.8 | 73.6 | ± | 13.9 | 72.1 | ± | 14.2 | 70.4 | ± | 12.7 |
| **eGFR _novel equation for Korean_ (mL/min/1.73m^2^)** |  |  |  |  |  |  |  |  |  |  |  |  |
| All | 83.8 | ± | 17.6 | 82.3 | ± | 17.5 | 86.0 | ± | 17.5 | 82.0 | ± | 17.7 |
| Males | 98.6 | ± | 9.6 | 98.8 | ± | 9.3 | 98.6 | ± | 9.4 | 97.9 | ± | 10.9 |
| Females | 70.1 | ± | 11.1 | 70.3 | ± | 11.1 | 70.2 | ± | 11.5 | 69.6 | ± | 10.6 |

Data are expressed as mean ± SD.

eGFR, estimated glomerular filtration rate; MDRD, Modification of Diet in Renal Disease; CKD-EPI, Chronic Kidney Disease Epidemiology Collaboration
