# Supplementary material for: Relationship between prehypertension and chronic kidney disease in middle-aged people in Korea: the Korean genome and epidemiology study
Source: BMC Public Health. 2012 Nov 9;12:960. doi: 10.1186/1471-2458-12-960 (PMC3549294; doi:10.1186/1471-2458-12-960)
Supplement: Additional file 2 — Prevalence of CKD according to the CKD-EPI equation. [file 1471-2458-12-960-S2.doc]

**Additional file 2.** Prevalence of CKD according to the CKD-EPI equation.

| **Variables** | **Total**  **(*n*=9508)** | | **BP category** | | | | | | ***P*-value** |
| --- | --- | --- | --- | --- | --- | --- | --- | --- | --- |
|  |  |  | **Normal BP**  **(*n*=3792)** | | **Prehypertension**  **(*n*=3873)** | | **Hypertension**  **(*n*=1843)** | |  |
| **eGFR _CKD-EPI_** |  |  |  |  |  |  |  |  |  |
| All (*n*=9508) |  |  |  |  |  |  |  |  |  |
| No-CKD | 8606 | (90.5) | 3638 | (95.9) | 3500 | (90.4) | 1468 | (79.7) | <0.001 |
| All CKD | 902 | (9.5) | 154 | (4.1) | 373 | (9.6) | 375 | (20.3) |  |
| Stages 1 and 2 | 185 | (20.5) | 41 | (26.6) | 80 | (21.4) | 64 | (17.1) |  |
| Stage 3 | 708 | (78.5) | 113 | (73.4) | 291 | (78.0) | 304 | (81.1) |  |
| Stage 4 | 9 | (1.0) | 0 | (0.0) | 2 | (0.5) | 7 | (1.9) |  |
| Males (*n*=4565) |  |  |  |  |  |  |  |  |  |
| No-CKD | 4410 | (96.6) | 1571 | (98.6) | 2090 | (96.6) | 749 | (92.8) | <0.001 |
| All CKD | 155 | (3.4) | 23 | (1.4) | 74 | (3.4) | 58 | (7.2) |  |
| Stages 1 and 2 | 112 | (72.3) | 20 | (87.0) | 54 | (73.0) | 38 | (65.5) |  |
| Stage 3 | 41 | (26.5) | 3 | (13.0) | 20 | (27.0) | 18 | (31.0) |  |
| Stage 4 | 2 | (1.3) | 0 | (0.0) | 0 | (0.0) | 2 | (3.4) |  |
| Females (*n*=4943) |  |  |  |  |  |  |  |  |  |
| No-CKD | 4196 | (84.9) | 2067 | (94.0) | 1410 | (82.5) | 719 | (69.4) | <0.001 |
| All CKD | 747 | (15.1) | 131 | (6.0) | 299 | (17.5) | 317 | (30.6) |  |
| Stages 1 and 2 | 73 | (9.8) | 21 | (16.0) | 26 | (8.7) | 26 | (8.2) |  |
| Stage 3 | 667 | (89.3) | 110 | (84.0) | 271 | (90.6) | 286 | (90.2) |  |
| Stage 4 | 7 | (0.9) | 0 | (0.0) | 2 | (0.7) | 5 | (1.6) |  |

Data are expressed as *n* (%) and tested by chi-square test at *P* < 0.05.
