# Supplementary material for: Relationship between prehypertension and chronic kidney disease in middle-aged people in Korea: the Korean genome and epidemiology study
Source: BMC Public Health. 2012 Nov 9;12:960. doi: 10.1186/1471-2458-12-960 (PMC3549294; doi:10.1186/1471-2458-12-960)
Supplement: Additional file 3 — Prevalence of CKD according to the MDRD equation with the Korean coefficient. [file 1471-2458-12-960-S3.doc]

**Additional file 3.** Prevalence of CKD according to the MDRD equation with the Korean coefficient.

| **Variables** | **Total**  **(*n*=9509)** | | **BP category** | | | | | | ***P*-value** |
| --- | --- | --- | --- | --- | --- | --- | --- | --- | --- |
|  |  |  | **Normal BP**  **(*n*=3792)** | | **Prehypertension**  **(*n*=3873)** | | **Hypertension**  **(*n*=1844)** | |  |
| **eGFR _MDRD with the Korean coefficient_** |  |  |  |  |  |  |  |  |  |
| All (*n*=9509) |  |  |  |  |  |  |  |  |  |
| No-CKD | 9255 | (97.3) | 3742 | (98.7) | 3768 | (97.3) | 1745 | (94.6) | <0.001 |
| All CKD | 254 | (2.7) | 50 | (1.3) | 105 | (2.7) | 99 | (5.4) |  |
| Stages 1 and 2 | 203 | (79.9) | 44 | (88.0) | 87 | (82.9) | 72 | (72.7) |  |
| Stage 3 | 44 | (17.3) | 6 | (12.0) | 17 | (16.2) | 21 | (21.2) |  |
| Stage 4 | 7 | (2.8) | 0 | (0.0) | 1 | (1.0) | 6 | (6.1) |  |
| Males (*n*=4566) |  |  |  |  |  |  |  |  |  |
| No-CKD | 4429 | (97.0) | 1573 | (98.7) | 2100 | (97.0) | 756 | (93.6) | <0.001 |
| All CKD | 137 | (3.0) | 21 | (1.3) | 64 | (3.0) | 52 | (6.4) |  |
| Stages 1 and 2 | 115 | (83.9) | 20 | (95.2) | 54 | (84.4) | 41 | (78.8) |  |
| Stage 3 | 19 | (13.9) | 1 | (4.8) | 10 | (15.6) | 8 | (15.4) |  |
| Stage 4 | 3 | (2.2) | 0 | (0.0) | 0 | (0.0) | 3 | (5.8) |  |
| Females (*n*=4943) |  |  |  |  |  |  |  |  |  |
| No-CKD | 4826 | (97.6) | 2169 | (98.7) | 1668 | (97.6) | 989 | (95.5) | <0.001 |
| All CKD | 117 | (2.4) | 29 | (1.3) | 41 | (2.4) | 47 | (4.5) |  |
| Stages 1 and 2 | 88 | (75.2) | 24 | (82.8) | 33 | (80.5) | 31 | (66.0) |  |
| Stage 3 | 25 | (21.4) | 5 | (17.2) | 7 | (17.1) | 13 | (27.7) |  |
| Stage 4 | 4 | (3.4) | 0 | (0.0) | 1 | (2.4) | 3 | (6.4) |  |

Data are expressed as *n* (%) and tested by chi-square test at *P* < 0.05.
