# Supplementary material for: Relationship between prehypertension and chronic kidney disease in middle-aged people in Korea: the Korean genome and epidemiology study
Source: BMC Public Health. 2012 Nov 9;12:960. doi: 10.1186/1471-2458-12-960 (PMC3549294; doi:10.1186/1471-2458-12-960)
Supplement: Additional file 4 — Prevalence of CKD according to the novel equation for Korean. [file 1471-2458-12-960-S4.doc]

**Additional file 4.** Prevalence of CKD according to the novel equation for Korean.

| **Variables** | **Total**  **(*n*=9509)** | | **BP category** | | | | | | ***P*-value** |
| --- | --- | --- | --- | --- | --- | --- | --- | --- | --- |
|  |  |  | **Normal BP**  **(*n*=3792)** | | **Prehypertension**  **(*n*=3873)** | | **Hypertension**  **(*n*=1844)** | |  |
| **eGFR _novel equation for Korean_** |  |  |  |  |  |  |  |  |  |
| All (*n*=9509) |  |  |  |  |  |  |  |  |  |
| No-CKD | 9279 | (97.6) | 3745 | (98.8) | 3777 | (97.5) | 1757 | (95.3) | <0.001 |
| All CKD | 230 | (2.4) | 47 | (1.2) | 96 | (2.5) | 87 | (4.7) |  |
| Stages 1 and 2 | 205 | (89.1) | 44 | (93.6) | 88 | (91.7) | 73 | (83.9) |  |
| Stage 3 | 22 | (9.6) | 3 | (6.4) | 7 | (7.3) | 12 | (13.8) |  |
| Stage 4 | 3 | (1.3) | 0 | (0.0) | 1 | (1.0) | 2 | (2.3) |  |
| Males (*n*=4566) |  |  |  |  |  |  |  |  |  |
| No-CKD | 4436 | (97.2) | 1573 | (98.7) | 2104 | (97.2) | 759 | (93.9) | <0.001 |
| All CKD | 130 | (2.8) | 21 | (1.3) | 60 | (2.8) | 49 | (6.1) |  |
| Stages 1 and 2 | 116 | (89.2) | 20 | (95.2) | 55 | (91.7) | 41 | (83.7) |  |
| Stage 3 | 13 | (10.0) | 1 | (4.8) | 5 | (8.3) | 7 | (14.3) |  |
| Stage 4 | 1 | (0.8) | 0 | (0.0) | 0 | (0.0) | 1 | (2.0) |  |
| Females (*n*=4943) |  |  |  |  |  |  |  |  |  |
| No-CKD | 4843 | (98.0) | 2172 | (98.8) | 1673 | (97.9) | 998 | (96.3) | <0.001 |
| All CKD | 100 | (2.0) | 26 | (1.2) | 36 | (2.1) | 38 | (3.7) |  |
| Stages 1 and 2 | 89 | (89.0) | 24 | (92.3) | 33 | (91.7) | 32 | (84.2) |  |
| Stage 3 | 9 | (9.0) | 2 | (7.7) | 2 | (5.6) | 5 | (13.2) |  |
| Stage 4 | 2 | (2.0) | 0 | (0.0) | 1 | (2.8) | 1 | (2.6) |  |

Data are expressed as *n* (%) and tested by chi-square test at *P* < 0.05.
